# Hemoptysis due to progressive scoliosis associated with congenital heart disease: a case report

**DOI:** 10.1186/s12891-022-05225-9

**Published:** 2022-03-18

**Authors:** Kohei Yamaguchi, Masashi Uehara, Hiroki Oba, Terue Hatakenaka, Shugo Kuraishi, Shota Ikegami, Takashi Takizawa, Ryo Munakata, Takayuki Kamanaka, Yoshinari Miyaoka, Kiyohiro Takigiku, Jun Takahashi

**Affiliations:** 1 Department of Orthopaedic Surgery, Shinshu University School of Medicine, 3-1-1 Asahi, Matsumoto, Nagano, 390-8621 Japan; 2 Department of Cardiology, Nagano Children’s Hospital, 3100 Toyoshina, Azumino, Nagano, 399-8288 Japan

**Keywords:** Scoliosis, Congenital heart disease, Hemoptysis, Pulmonary vein stenosis, Spinal correction

## Abstract

**Background:**

Patients with congenital heart disease (CHD) are associated with an increased incidence of scoliosis, often with severe progression. We report a case of hemoptysis caused by rapid scoliosis progression subsequent to surgery for CHD that was successfully managed by surgical curve correction following coil embolization.

**Case presentation:**

A 14-year-old girl with scoliosis had undergone open heart surgery for CHD at the age of 1 year. She was first noted to have scoliosis at 12 years of age, which began to progress rapidly. At age 13, her main thoracic curve Cobb angle was 46°, and hemoptysis with high pulmonary vein pressure due to vertebral rotation was detected. Nine months after coil embolization, she received posterior spinal fusion from T5 to L2 for scoliosis correction. Postoperatively, her pulmonary vein diameter was enlarged, with no detectable signs of hemoptysis.

**Conclusions:**

We encountered a case of hemoptysis caused by advanced scoliosis after cardiac surgery that was successfully treated by correction of the scoliotic curve following coil embolization. Patients with secondary scoliosis after surgery for CHD should be carefully monitored for the possibility of cardiovascular system deterioration.

## Background

Patients with congenital heart disease (CHD) are associated with an increased risk of scoliosis, with a reported incidence of 10–19% [1]. This rate is considerably higher (42.4%) in patients who undergo cardiac surgery before the age of 1 year [2]. The association between scoliosis and CHD is multifactorial since children with CHD are more likely to have fetal abnormalities involving the spine and thorax as well as a predisposition to thoracic scoliosis associated with thoracic surgery [3–6]. In patients with CHD, scoliosis is common following median sternotomy [7]. The scoliosis that develops after surgery for CHD is often severe and progressive and can diminish activities of daily living and respiratory function. Advances in cardiac treatment have improved the prognosis of patients with CHD, leading to an increase in the frequency of scoliosis correction surgeries in this population [8]. However, spinal care information is sparse. We herein report an unusual case of hemoptysis improvement after coil embolization and posterior spinal fusion in a scoliosis patient with prior surgery for CHD.

## Case presentation

**Fig. 1:**
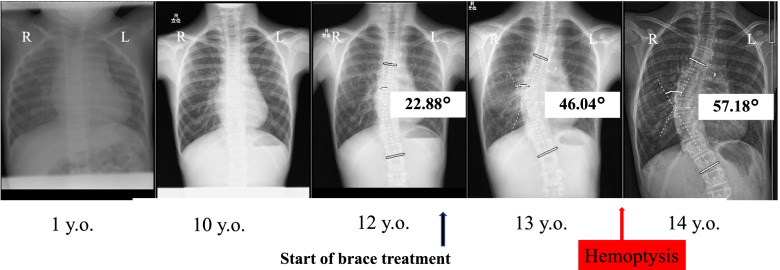
Changes in preoperative radiographs. At age 13, her main thoracic curve had a Cobb angle of approximately 46 degrees, with episodes of hemoptysis

**Fig. 2:**
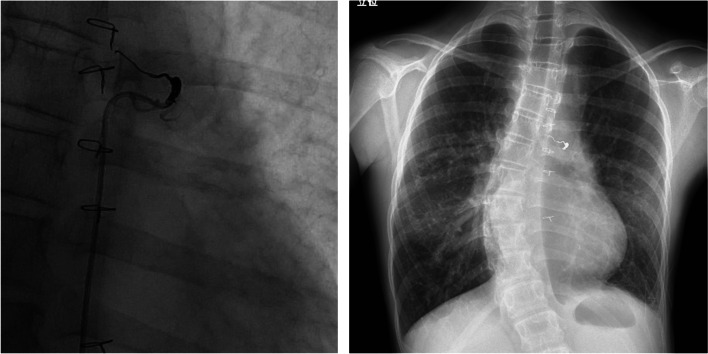
Coil embolization. Collateral blood flow from the left bronchial artery to the left pulmonary vein necessitated coil embolization

A 14-year-old girl with scoliosis following cardiac surgery for CHD early in life presented to our hospital for hemoptysis treatment. She had congenital heart malformations, including bilateral right ventricular origin of the aorta and an atrial septal defect, and had undergone open-heart surgery at the age of 1 year. During the surgery, median sternotomy was performed to reach the heart, although neither the ribs nor the vertebral bodies were affected. She had no other congenital diseases. At 12 years of age, she was first noted to have scoliosis with a main thoracic curve Cobb angle of 22 degrees and commenced brace treatment (Fig. 1a). At the age of 13, however, her main thoracic curve Cobb angle had progressed to 46 degrees and she suffered from hemoptysis, for which coil embolization was performed (Figs. 1 and 2). On presentation 9 months later, her height was 143.7 cm, her weight was 27.6 kg, her BMI was 13.5 kg/m^2^, and she had not had her first menstruation. Her Risser grade was 0 and she exhibited closure of the triradiate cartilage.

**Fig. 3:**
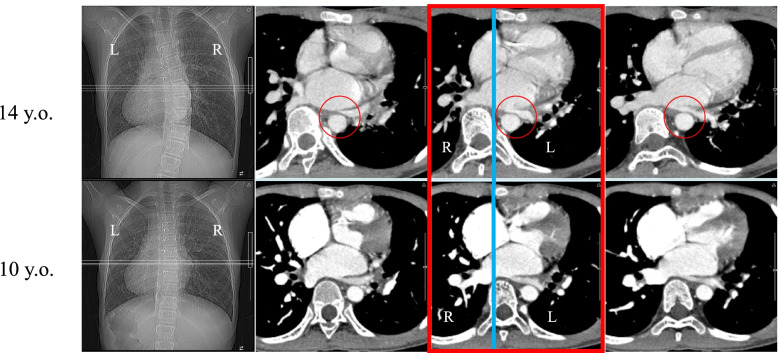
Preoperative contrast CT. Lateral deviation and right anterior rotation of the T8 vertebral body were evident. The descending aorta appeared to be draining in close proximity to the left pulmonary vein (circles)

Preoperatively, her main thoracic curve was T5-L1 with a Cobb angle of 57 degrees (Fig. 1). Simple magnetic resonance imaging of the chest revealed a right-to-left difference in pulmonary blood flow ratio of 10:1. Contrast-enhanced computed tomography (CT) of the chest showed that the left pulmonary vein was being compressed by the deviated descending aorta (Fig. 3). Echocardiography indicated no atrioventricular valve regurgitation or systolic or diastolic capacity abnormalities in either ventricle. Cardiac catheterization revealed that pulmonary artery wedge pressure, which has been reported to correlate with pulmonary venous pressure, was higher on the left side (23 mmHg) than on the right side (17 mmHg), suggesting that her hemoptysis was caused by pulmonary congestion from stenosis of the left pulmonary vein (hemoglobin [Hb]: 10.5 g/dL).

Based on the above results, her left pulmonary vein stenosis was presumed to have resulted from compression of the left pulmonary vein by the descending aorta from lateral deviation and right anterior rotation of the vertebrae due to the progression of scoliosis on contrast-enhanced CT since the age of 10 years (Fig. 3). Our hospital policy was to prioritize corrective scoliosis surgery to release the vein compression.

**Fig. 4:**
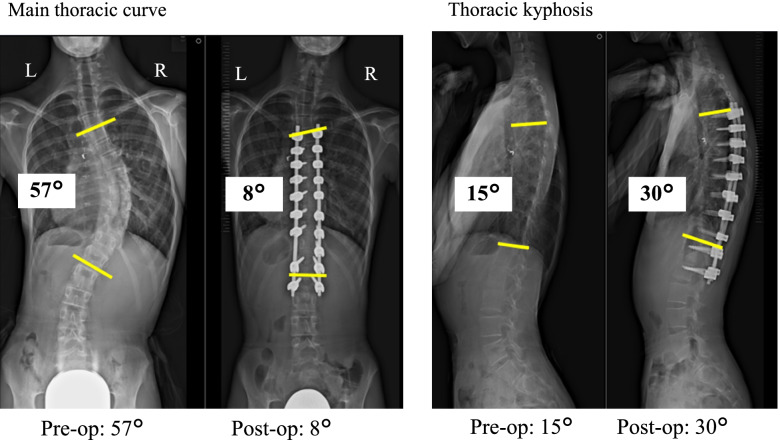
Pre- and postoperative radiographs. Radiographs showed that the correction rate of the main thoracic curve was 72% and thoracic kyphosis angle had increased by 15 degrees

**Fig. 5:**
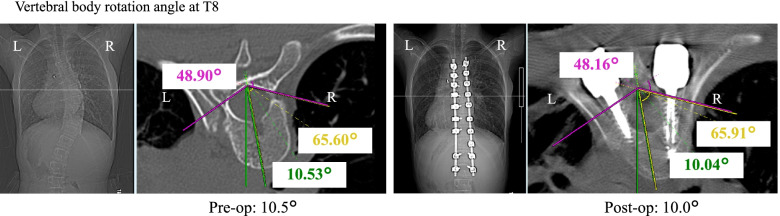
Pre- and postoperative CT. CT indicated that vertebral body rotation angle was slightly decreased from 10.5 degrees to 10.0 degrees at T8, which was the apical vertebra

**Fig. 6:**
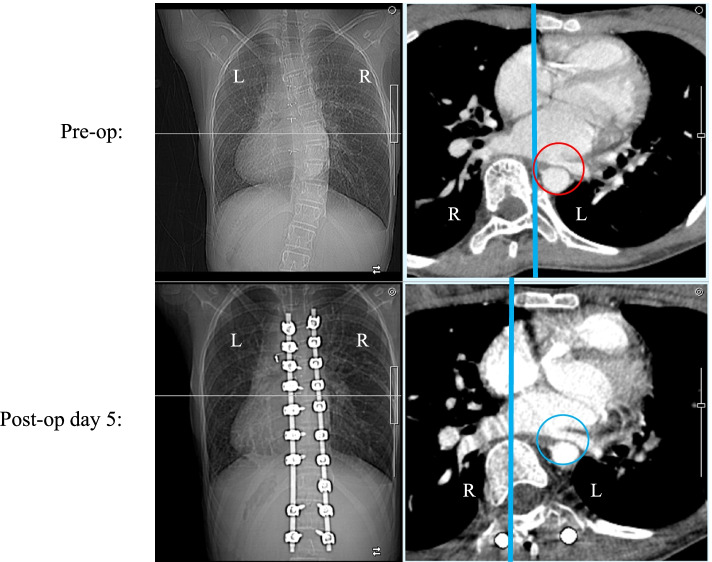
Changes in pre- and postoperative contrast CT. A midline and posterior shift of the T8 vertebral body relative to the sternum (blue circle) as compared with preoperative images (red circle)

The patient first received coil embolization 9 months prior to posterior spinal fusion from T5 to L2 using the vertebral coplanar alignment technique [9] with spinous process preservation. After screw insertion, corrective force was first applied to the convex, non-stenotic vessel side to correct the scoliosis and rotational deformity simultaneously. Operation time was 238 min and blood loss was 150 mL. Her main thoracic curve Cobb angle improved to 8 degrees (72% correction rate) and thoracic kyphosis angle was ameliorated from 15 degrees to 30 degrees (Fig. 4). Regarding spinal rotation, vertebral body rotation angle [10] was slightly decreased from 10.5 degrees to 10.0 degrees at the apical T8 vertebra (Fig. 5). Contrast-enhanced CT on the sixth postoperative day showed a midline and posterior shift of the vertebral bodies relative to the sternum as compared with preoperative images, suggesting that the aorta was exerting less pressure on the left pulmonary vein (Fig. 6). The patient could walk continuously for 70 m on the third postoperative day and climb stairs on the seventh postoperative day, with no detectable shortness of breath or other symptoms indicating pulmonary congestion. On postoperative day 13, echocardiography confirmed an enlarged left pulmonary vein diameter from 4 mm preoperatively to 7 mm after surgery (Table 1). She returned home on postoperative day 14 (Hb: 11.1 g/dL), with no hemoptysis recurrence in the 6 months since discharge.

**Table 1:** Pre- and postoperative pulmonary vein diameter expansion confirmed by echocardiography

	Left pulmonary artery wedge pressure	Left pulmonary vein diameter
Pre-op	23 mmHg	4 mm
Post-op	22 mmHg	7 mm

This is a case report. The institutional ethics review board of authors’ facility has confirmed that no ethical approval is required. Consent was obtained from the patient’s parents for participation in this study.

## Discussion

Severe scoliotic deformity with rotation of the spine often occurs after cardiac surgery for CHD [1, 2]. However, the exact mechanism of scoliosis development remains uncertain. Van Biezen et al. reported that all patients who underwent left thoracotomy later exhibited secondary scoliosis [11]. On the other hand, Reckles et al. observed no increase in the incidence of scoliosis after CHD surgery [12]. Years after cardiac surgery for CHD in the present case, the patient displayed severe and rapidly progressing scoliosis, which adversely affected her circulation and caused hemoptysis. Coil embolization followed by surgical correction of the scoliotic deformity resulted in an improvement in her circulatory dynamics.

The mechanism of our patient’s hemoptysis was thought to be pulmonary vein stenosis, with pulmonary congestion due to increased pulmonary venous pressure resulting in intralobular hemorrhage and hemoptysis. Indeed, cardiac ablation therapy performed in the left atrium can become complicated by stenosis of the left pulmonary vein and cause subsequent pulmonary congestion and hemoptysis [13, 14].

In the field of orthopedics, emergency thoracoplasty for the treatment of scoliosis in patients with funnel chest can be lifesaving [15, 16], suggesting that scoliosis itself and its correction may significantly alter the anatomy of organs within the thorax [17] and affect circulatory dynamics. In this case, the progression of scoliosis and vertebral rotation exacerbated pulmonary vein stenosis and increased pulmonary vein pressure, which resulted in hemoptysis. After correction surgery, the diameter of the left pulmonary vein was enlarged and hemoptysis was no longer observed. However, pulmonary artery wedge pressure, which has been correlated with pulmonary venous pressure, did not change between before and after the surgery; a limitation of this report was that we could not conclusively determine whether the cause of the increased pulmonary venous pressure was pulmonary vein stenosis (Table 1).

We routinely consider a Cobb angle of 45 degrees or more as an indication for surgery in adolescent idiopathic scoliosis. For post-cardiac surgery scoliosis and syndromic scoliosis, however, a Cobb angle of 60 degrees or more is judged to require surgery due to the high risk of surgical complications. In the present case, surgery was initially withheld because of the limited hemoptysis and the risk of surgery itself from complicating heart disease. As the scoliosis progressed, her hemoptysis worsened and we finally opted for surgery. Based on the course of this case, surgeons should consider earlier scoliosis treatment when hemoptysis due to pulmonary hypertension is suspected.

## Conclusions

We report a case of hemoptysis caused by advanced scoliosis following cardiac surgery that improved after scoliosis correction following coil embolization. The release of pulmonary vein compression by scoliosis surgery was presumed to be the reason for the hemoptysis resolution. When examining patients with scoliosis secondary to CHD surgery, special attention should be paid to the possibility of circulatory deterioration associated with scoliosis progression.

## Data Availability

The datasets used and/or analyzed during the current study are available from the corresponding author on reasonable request.

## Abbreviations

CHD: Congenital heart disease
CT: Computed tomography
Hb: Hemoglobin
